# Digital Radiography with Computerized Conventional Monitors Compared to Medical Monitors in Vertical Root Fracture Diagnosis

**Published:** 2013-01-20

**Authors:** Maryam Tofangchiha, Mamak Adel, Mahin Bakhshi, Mahsa Esfehani, Pantea Nazeman, Mojgan Ghorbani Elizeyi, Amir Javadi

**Affiliations:** 1 Department of Radiology, Dental School, Qazvin University of Medical Sciences, Qazvin, Iran; 2 Department of Endodontics, Dental School, Qazvin University of Medical Sciences, Qazvin, Iran; 3 Department of Oral Medicine , Dental School, Shahid Beheshti University of Medical Sciences, Tehran, Iran; 4 Department of Oral Medicine, Dental School, Qazvin University of Medical Sciences, Qazvin, Iran; 5 Research center, Dental School, Qazvin University of Medical Sciences, Qazvin, Iran; 6 Department of Biostatistics, Dental School, Qazvin University of Medical Sciences, Qazvin, Iran

**Keywords:** Diagnostic Imaging, Diagnosis, Digital Dental Radiology, Monitor, Tooth Fractures, Radiography

## Abstract

**Introduction:**

Vertical root fracture (VRF) is a complication which is chiefly diagnosed radiographically. Recently, film-based radiography has been substituted with digital radiography. At the moment, there is a wide range of monitors available in the market for viewing digital images. The present study aims to compare the diagnostic accuracy, sensitivity and specificity of medical and conventional monitors in detection of vertical root fractures.

**Material and Methods:**

In this in vitro study 228 extracted single-rooted human teeth were endodontically treated. Vertical root fractures were induced in 114 samples. The teeth were imaged by a digital charge-coupled device radiography using parallel technique. The images were evaluated by a radiologist and an endodontist on two medical and conventional liquid-crystal display (LCD) monitors twice. Z-test was used to analyze the sensitivity, accuracy and specificity of each monitor. Significance level was set at 0.05. Inter and intra observer agreements were calculated by Cohen’s kappa.

**Results:**

Accuracy, specificity and sensitivity for conventional monitor were calculated as 67.5%, 72%, 62.5% respectively; and data for medical grade monitor were 67.5%, 66.5% and 68% respectively. Statistical analysis showed no significant differences in detecting VRF between the two techniques. Inter-observer agreement for conventional and medical monitor was 0.47 and 0.55 respectively (moderate). Intra-observer agreement was 0.78 for medical monitor and 0.87 for conventional one (substantial).

**Conclusion:**

The type of monitor does not influence diagnosis of vertical root fractures.

## 1. Introduction

Analogue radiography is being replaced by digital imaging, mainly due to its convenience [1-4]. One of the essential tools for viewing digital images is a monitor [5]. There is a great range of monitors available in the market with various contrast degree and sharpness [6]. As a result of rapid development in this system, Cathode-ray-tube (CRT) monitors have been replaced by liquid-crystal display (LCD) monitors and these monitors possess some advantages over CRT monitors, such as elimination of peripheral distortion artifacts and excellent spatial resolution [7, 8].

Vertical root fracture (VRF) is a complication that can occur during root canal therapy which is characterized by a fracture line through the long axis of the tooth, originating from the apical end and propagating to the crown. The incidence of this complication ranges from 11%-20%. The differential diagnosis of VRF might be difficult due to lack of specific symptoms and radiologic features [9, 10]. It is difficult to detect fractures in routine conventional radiography. A hairline radiolucent line [11] or a ‘‘halo’’ appearance, which is a combined periapical and perilateral radiolucency in one or both sides of the root, lateral periodontal radiolucency along the side of the root, and angular radiolucency from the crestal bone confined to the root side are radiographic features of vertically fractured teeth [10]. Diagnosis of vertical root fractures are confined due to several reasons such as narrowness of the fracture line, location and amount of extension, super imposition of other structures on the root such as lamina dura and periodontal ligament [12].

Recently, medical LCD monitors have been released to the market; they are assumed to posses the ability of displaying fine structures in details, yet manufacturers of these monitors claim that comparing to conventional LCD monitors, these displays possess higher accuracy. On the other hand, the medical monitors available in the market are far more expensive in comparison with generally used ones. Therefore, this study aimed to compare the diagnostic accuracy of medical and conventional monitors in detection of vertical root fractures.

## 2. Material and Methods

Two hundred and twenty eight human single-rooted extracted teeth, due to periodontal disease or for orthodontic treatments, with closed apices were used in this experimental in vitro research. The teeth were disinfected and preserved in 4% thymol solution for one week. The crowns were decoronated 2 mm above the CEJ by a paper disk. Buccal surfaces of teeth were marked by ink and then the teeth were mounted in the red compound impression material. Access cavities were prepared and the teeth were instrumented according to passive step back technique up to master file #40. Root canals were flared to a file #70. Subsequently, the samples were numbered and divided into two groups: a control group with no fracture of 114 teeth and a test group of 114 fractured teeth. Vertical root fractures were induced in the study group according to the method described by Monagham et al. [13]. For this reason, a 60º beveled tip conical wedge was apically driven into the root by a bur until there was a sharp cracking sound. After inducing fractures in experimental samples, samples of both groups were imaged with Kodak E Speed No2 periapical films by parallel technique in the facio-lingual view while the long axes of the teeth were parallel to the receptors. The images were obtained by a fifth generation Radio Vision Graphy CCD receptor (Trophy, France) and all radiographic exposures were made by Planmeca dental X-ray unit (Planmeca, Finland) at 63 kVp, 8 mA and 0.1 s and the focus object distance was 20 cm.

After imaging, the images were randomly numbered and displayed on a conventional 19 inch color LCD monitor (Syncmaster BW1953, Samsung, Korea) with a resolution of 1440×900 pixels, and a 19-inch color LCD medical monitor (Flexscan MX190S, Eizo, Japan) with a resolution of 1280×1024 pixels.

Blind evaluation of images was performed by two observers, one oral and maxillofacial radiologist and one endodontist; each held at least 5 years of experience in their fields. The observers categorized the images as “tooth with fracture” or “tooth with no fracture”. The data analysis was performed by SPSS V15. Inter and intra observer agreements were calculated by Cohen’s kappa and were ranked accordingly [14].

## 3. Results

Accuracy, sensitivity and specificity of the monitors are illustrated in Table 1. There was no statistically significant difference between accuracy, sensitivity and specificity of two types of monitors.

**Table 1.** Accuracy, sensitivity and specificity of the Monitors

Monitor	Sensitivity	Specificity	Accuracy
**Conventional**	62.5%	72%	67.5%
**Medical**	68%	66.5%	67.5%
**P-value**	0.2	0.22	1

The intra observer agreement was calculated by kappa coefficient which demonstrates 0.78 for medical monitors and 0.87 for conventional monitors. This calculation suggested that intra observer agreement for conventional and medical monitors was substantial (Table 2). Inter observer agreement was evaluated by Kappa correlation moderate, reporting 0.470 for conventional monitors and 0.550 for medical monitors (Table 3).

**Table 2.** Intra observer agreement in the medial and conventional monitors

Monitor	Observer	Kappa
**Conventional**	1	0.561
	2	0.874
**Medical**	1	0.581

**Table 3.** Inter observer agreement in the medial and conventional monitors

Monitor	Kappa
**Medical grade**	0.550
**Conventional**	0.470

## 4. Discussion

Radiographic assessment is an important procedure in vertical root fracture diagnosis. It is postulated that diagnosis of vertical root fractures is confined by displaying systems (conventional or digital) recruited in this procedure [15, 16]. This study aimed to compare two monitors with two different applications, one conventional monitor which is routinely available in the market and one medical monitor which is 10 times more costly. In studies conducted by Ilguy et al., Isidor et al. and Halme et al., medical monitors demonstrated to be more accurate in caries detection though this finding was not statistically significant, therefore it may not be worthwhile to invest in a monitor that is so costly [17-19]. Similar conclusion is made in studies of Esmaeili et al. and Ludlow et al. as well [20,21].

In this study, the accuracy of two monitors was similar in detection of vertical root fractures (67%). Medical monitor was found to be more sensitive and the specificity was higher in conventional monitors however these differences were not statistically significant. In the past, the major drawback of conventional monitors was their low luminance, so that it was harder to see the entire grayscale (from black to white) in an image [22] while in this study the monitors possessed similar luminance (300 cd/m2) therefore this could be an appropriate explanation for not detecting a significant difference between the accuracy of both monitors. On the other hand, this finding concurs with the results of Ilguy et al., Isidor et al. and Halme et al. [17-19]. In accordance with Hitomi et al.’s research on caries detection by two monitors, no difference was demonstrated in detecting caries [23].

Observer’s agreement in detecting vertical root fractures by two monitors was assessed in this study as well. Results demonstrated higher internal agreement (reliability) comparing to inter observer agreement, which was similar to results obtained by Halme and Naitoh [18, 24].

Intra observer agreement correlation was 0.78 and 0.87 for medical and conventional monitors respectively, thus both results were categorized substantial agreement and both monitors possessed similar accuracy in this variable. External agreement was calculated at 0.55 and 0.47 for medical and conventional monitors, respectively which can be graded as moderate inter observer agreement.

As mentioned in Ludlow’s study, in order to eliminate factors affecting the results, the viewing angle must be 90 degrees between the monitor and horizon [21]. The observers of the present study considered this fact when evaluating the images while to our knowledge there was no consideration on this fact in Halme’s and Hitomi’s studies [18, 23].

The previous studies were generally conducted on medical monochrome displays and color LCD displays. One of the major advantages of color LCDs is their ability to show color images [22]. In this study a medical color display was recruited to eliminate color as a variable.

According to various other medical studies which looked at brain CT [25], radiography of wrist fractures [26, 27], computed radiographs of the hands in early rheumatoid arthritis [28], and chest radiographs in interstitial lung disease [29] with different displays, no significant difference was detected between the monitors, concurring with our study.

## 5. Conclusion

Results of the present study suggest that the conventional and medical monitor possessed similar accuracy, specificity and sensitivity in detection of vertical root fractures. The type of monitor is not a determining factor in diagnostic accuracy of VRF and the conventional affordable color LCD monitors available in the market seem to be adequate.
